# miR-10a-3p modulates adiposity and suppresses adipose inflammation through TGF-β1/Smad3 signaling pathway

**DOI:** 10.3389/fimmu.2023.1213415

**Published:** 2023-06-02

**Authors:** Sonia Kiran, Mousumi Mandal, Ahmed Rakib, Amandeep Bajwa, Udai P. Singh

**Affiliations:** ^1^Department of Pharmaceutical Sciences, College of Pharmacy, The University of Tennessee Health Science Center, Memphis, TN, United States; ^2^Department of Surgery, College of Medicine, The University of Tennessee Health Science Center, Memphis, TN, United States

**Keywords:** inflammation, obesity, adipocytes, TGF-β1, microRNA

## Abstract

**Background:**

Obesity is a multifactorial disease characterized by an enhanced amount of fat and energy storage in adipose tissue (AT). Obesity appears to promote and maintain low-grade chronic inflammation by activating a subset of inflammatory T cells, macrophages, and other immune cells that infiltrate the AT. Maintenance of AT inflammation during obesity involves regulation by microRNAs (miRs), which also regulate the expression of genes implicated in adipocyte differentiation. This study aims to use *ex vivo* and *in vitro* approaches to evaluate the role and mechanism of miR-10a-3p in adipose inflammation and adipogenesis.

**Methods:**

Wild-type BL/6 mice were placed on normal (ND) and high-fat diet (HFD) for 12 weeks and their obesity phenotype, inflammatory genes, and miRs expression were examined in the AT. We also used differentiated 3T3-L1 adipocytes for mechanistic *in vitro* studies.

**Results:**

Microarray analysis allowed us to identify an altered set of miRs in the AT immune cells and Ingenuity pathway analysis (IPA) prediction demonstrated that miR-10a-3p expression was downregulated in AT immune cells in the HFD group as compared to ND. A molecular mimic of miR-10a-3p reduced expression of inflammatory M1 macrophages, cytokines, and chemokines, including transforming growth factor-beta 1 (TGF-β1), transcription factor Krüppel-like factor 4 (KLF4), and interleukin 17F (IL-17F) and induced expression of forkhead box P3 (FoxP3) in the immune cells isolated from AT of HFD-fed mice as compared to ND. In differentiated 3T3-L1 adipocytes, the miR-10a-3p mimics also reduced expression of proinflammatory genes and lipid accumulation, which plays a role in the dysregulation of AT function. In these cells, overexpression of miR-10a-3p reduced the expression of TGF-β1, Smad3, CHOP-10, and fatty acid synthase (FASN), relative to the control scramble miRs.

**Conclusion:**

Our findings suggest that miR-10a-3p mimic mediates the TGF-β1/Smad3 signaling to improve metabolic markers and adipose inflammation. This study provides a new opportunity for the development of miR-10a-3p as a novel therapeutic for adipose inflammation, and its associated metabolic disorders.

## Graphical Abstract

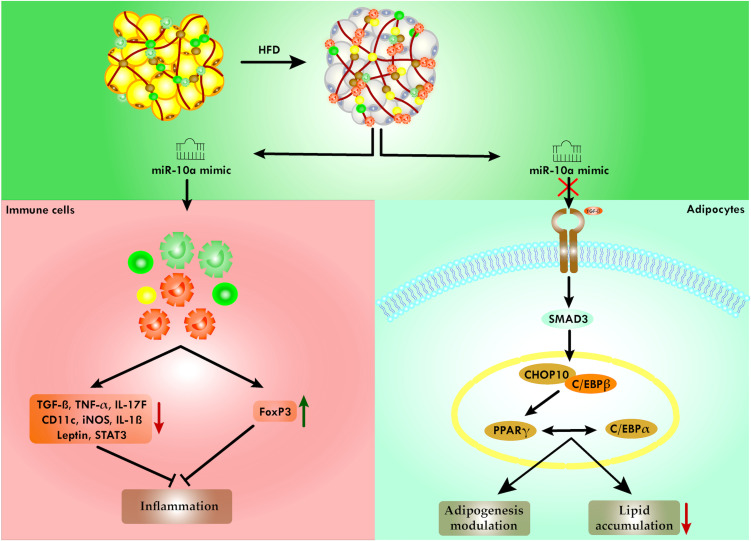

## Introduction

Obesity is a multifactorial disease characterized by a body mass index (BMI) ≥ 30, excessive lipid accumulation in adipose tissue (AT), and an imbalance in energy intake and expenditure (1). The global epidemic of obesity is one of the major healthcare challenges in the modern world (2). It is estimated that half of all Americans will be obese by 2030 and that women will be disproportionately affected (3). Obesity is strongly associated with several diseases including insulin resistance, type 2 diabetes mellitus (T2DM), cardiovascular disease, fatty liver, and cancer (4). The main etiological factors for the development of obesity include diet, genetics, epigenetics, hormonal imbalance, and physical activity. During obesity, the generation of free fatty acids (FA) causes lipotoxicity in non-adipose tissues (5), and dysregulation of the immune cells contributes to low-grade chronic inflammation (6). Numbers of AT-resident proinflammatory M1 macrophages, T helper cells 1 (Th1), and Th17 cells increase (7) during obesity. These cells secrete tumor necrosis factor α (TNF-α), interleukin 6 (IL-6), IL-1β, and IL-17, which aberrantly regulate adipocyte biology (8–10). During obesity, AT is expanded by hypertrophy and hyperplasia through increased volume and recruitment of preadipocytes, respectively, although preadipocyte differentiation is impaired (11). TNF-α and IL-6 inhibit preadipocyte differentiation and induce hypertrophy (12). These hypertrophic adipocytes secrete monocyte chemoattractant protein 1 (MCP-1), leptin, TNF-α, and IL-6, which attract more immune cells to the AT and induce an inflammatory response (13, 14). Thus, under obese conditions, a vicious cycle of chronic inflammation and ectopic adipogenesis is maintained between immune cells and adipocytes in the AT. In turn, these serve to increase the susceptibility to infection and reduce the immune response, which is the crucial cause of many other inflammatory and autoimmune diseases. The current interventions are insufficient to prevent morbidity and mortality, in part, because the early triggers and signals that establish and sustain AT inflammation in obesity remain elusive. Thus, there is an urgent need to demonstrate the pathogenesis and crosstalk between adipocyte and immune cells to design an effective therapeutic strategy to combat AT inflammation and adiposity.

Cytokines, hormones, and signaling pathways are important regulators of obesity. For example, TNF-α is critical for the advancement of obesity (15). The signal transducer and activator of transcription 3 (STAT-3) initiate signaling pathways involved in the differentiation of Th17 cells (16), which are associated with obesity-induced inflammation. Obese individuals exhibit increased infiltration of Th17 cells into the AT (17). The novel regulator of macrophage polarization Krüppel-like factor 4 (KLF4) also controls adipogenesis (18). The master regulator of adipocyte biology, peroxisome proliferator-activated receptor gamma (PPAR-γ) is involved in adipocyte differentiation and glucose metabolism. PPAR-γ deficiency in macrophages results in increased obesity-induced AT inflammation (19). CCAAT/enhancer-binding protein (C/EBP) and C/EBP homologous protein 10 (CHOP-10) intricately regulate adipogenesis (20). Furthermore, the expression of fatty acid synthase (FASN) is positively correlated with obesity and related metabolic dysregulation (21). The leptin and transforming growth factor-beta1 (TGF-β1)/Smad3 signaling pathways are also critically associated with the development of obesity (22). Likewise, overexpression of TGF-β1/Smad protein is positively correlated with obesity (23). Thus, determining how these signaling pathways are intricate during AT inflammation is crucial to fight against AT inflammation and obesity.

Obesity is also regulated by microRNAs (miRs), small (approximately 22 nucleotides), and single-stranded noncoding RNAs that function by binding mRNAs and inhibiting their translation (24). miRs regulate the changes in obese AT, can accelerate or inhibit adipocyte differentiation and chronic inflammation, and may serve as targets for future therapeutic development (25). Dysregulation of miRs in metabolic tissues, including muscles, liver, and AT, contributes to obesity-related diseases (26). Interestingly, we noticed that expression of miR-10a is downregulated in the AT immune cell population isolated from mice fed on an HFD, relative to its levels in the same cells isolated from mice fed on a ND. miR-10a regulates the expression of TGF-β1 in renal and hepatic fibrosis (27, 28) and the miR-10a/b and TGF-β1 pathways interact through a negative feedback loop during ovarian granulosa cell development (29). However, how miR-10a-3p mediates TGF-β1/Smad3 expression, adipogenesis, and AT inflammation *via* crosstalk between immune cells and adipocytes is not well established in the context of obesity.

Our approach to better understanding the role of miR-10a-3p in this process involves the use of miR mimics and antagomiRs (30). Synthetic miR mimics have structural and functional homology with endogenous miRs. When transfected into cells, miR mimics can revive the function of their cognate endogenous miR (31). Similarly, synthetic antagomiRs silence the function of their cognate endogenous miRs. Although miR mimics and antagomiRs have been tested as therapeutic tools for obesity, T2DM, insulin resistance, and cardiovascular disease, they have met with limited success so far (32–34).

Thus, our goal in this study was to determine the mechanism by which miR-10a-3p overexpression mediates AT inflammation, adiposity, and obesity. Our results suggest that the miR-10a-3p mimic reduces the expression of leptin, STAT-3, adipogenic markers, TGF-β1, Smad3, FASN, C/EBPα, and CHOP-10. The miR-10a-3p mimic significantly reduced levels of various inflammatory cytokines and blocked excess lipid accumulation in adipocytes through the TGF-β1/Smad3 pathway during obesity. Thus, the miR-10a-3p overexpression serves as a potential therapeutic tool to modulate the function of adipose tissue and thereby control obesity.

## Materials and methods

### Animals and ethics statement and study design

All animal experimentation was performed under protocols (20-0162) approved by the University of Tennessee Health Science Center (UTHSC) Institutional Animal Care and Use Committee (IACUC). Wild-type (WT) C57BL/6J male mice (7 weeks old) were purchased from Jackson Laboratories (Bar Harbor, ME, USA) and housed in a pathogen-free animal facility at UTHSC with normal 12/12 h light/dark cycles. The mice were housed for a week to acclimatize them to the animal facility before starting the experiment, then randomly divided into two experimental groups that each contained 6 mice (n=6). The groups of now 8-week-old mice were fed either a 10% kcal normal diet (ND; D12450J, Research Diets, New Brunswick, NJ) or a 60% kcal high-fat diet (HFD; D12492, Research Diets) for 12 weeks. Their body weight was measured weekly, while their blood glucose and insulin levels were monitored before euthanasia.

### Quantification of AT adipokines by Bio-Plex assay

The 7-plex MILLIPLEX® Mouse Adipocyte Magnetic Bead Panel (Endocrine Multiplex Assay, MADCYMAG-72K, Millipore Sigma) allows simultaneous quantification of mouse adipose tissue (AT) analytes adiponectin, IL-6, leptin, MCP-1, plasminogen activator inhibitor-1 (PAI-1), Resistin, and TNF-α. Epididymal AT was collected from mice fed on ND and HFD after sacrifice, tissue lysates were prepared using the manufacturer’s protocol, and cell debris was pelleted by centrifugation. In brief, assay buffer was added to each well of a 96-well plate, incubated at room temperature (RT) for 10 min, and removed. A serially diluted mouse adipokine standard, a quality control, or an undiluted experimental sample was added to a parallel well, and assay buffer was added, followed by a mixture of the 7 specific antibody-coated beads. After overnight incubation at 4°C with shaking, the contents of each well were gently removed and the plate was washed. Detection antibodies were added, the plate was incubated at RT for 30 min, streptavidin-phycoerythrin was added, and the plate was incubated at RT for another 30 min. Unbound contents were removed by washing. The signal was detected using a Bio-Plex instrument with Luminex™ xMAP technology (Bio-Rad Laboratories, Austin, TX, USA), and the data were analyzed using Bio-Plex Manager software (BioRad).

### Fixation and hematoxylin & eosin staining of AT

AT was fixed in 4% paraformaldehyde, embedded in paraffin blocks, cut into 6 μm thick sections, and collected on a glass slide. Paraffin was removed by xylene and the tissue was rehydrated gradually by lowering alcohol gradation and stained with hematoxylin and eosin (H&E). Excess stain was removed by washing, the tissue was dehydrated with higher alcohol gradation, and the stained tissue was mounted permanently and used for imaging.

### Imaging and morphometric quantification

Microphotographs of H & E stained tissues were captured with an Olympus BX43 bright field microscope equipped with a 10X objective and an 8.9-megapixel CCD color digital camera (DP28). Cell morphological parameters like cell area and perimeter were quantified from 100X magnified images from each study group after pixel to μm conversion using ImageJ software (NIH) (Number of cells, n = 100/group)

### Microarray analysis of miRs

The epididymal AT collected from the ND and HFD mice was used to prepare AT single cells suspension. Briefly, AT was processed in MACS™ C tubes (130-096-334, Miltenyi Biotec USA, Gaithersburg, MD) and a gentle MACS™ Dissociator (130-093-235, Miltenyi Biotec) following the manufacturer’s protocol for the adipose tissue dissociation kit (130-105-808, Miltenyi Biotec). The resulting cell suspension was passed through a 100 mM cell strainer to generate a single-cell suspension and AT immune cells were separated from a stromal vascular fraction (SVF) using CD45 MicroBeads (130-052-301, Miltenyi Biotec) and a MACS™ magnetic separator. Total RNA, including miRs, from the AT immune cell population was isolated as described by the manufacturer’s protocol, and samples were stored at -80^0^C. For miR microarray analysis, the samples were sent to the John Hopkins University sequencing core facility, where the RNA was hybridized to an Affymetrix GeneChip high-throughput miR array containing 609 murine probes (Affymetrix, Santa Clara, CA). The resulting array data were analyzed using hierarchical clustering with Ingenuity pathway analysis (IPA) software (Qiagen; www.ingenuity.com) to identify molecular pathways that might be altered by single or multiple miR target genes. This analysis compares each set of miRs to all available pathways in the database and assigns priority scores based on the predicted strength of the miRs interaction with components of the target pathway.

### *Ex vivo* treatment of AT immune cells with miR-10a-3p mimic

The AT immune cells isolated from the AT of mice fed on HFD for 12 weeks, as described above in our earlier work, were seeded in 12-well plates at a density of 0.5x10^6^/well in RPMI medium. Cells were treated with 5 μM of the miR-10a-3p mimic MIM-hsa-miR-10a-3p (AUM Biotech, Philadelphia, PA) or control scrambled miR and incubated for 24 h at 37°C, 95% humidity in a 5% CO_2_ incubator.

### *In vitro* treatment of differentiated 3T3-L1 adipocytes with miR-10a-3p mimic

The 3T3-L1 preadipocyte cell line (ATCC-CL-173) was cultured in DMEM supplemented with 10% fetal bovine serum (FBS) at 37°C, 95% humidity, and 5% CO_2_ and induced to differentiate to adipocyte as described our previous work (35). In brief, the 3T3-L1 were induced to differentiate into adipocytes by incubation for 48 h in DMEM/10% FBS supplemented with 0.5 mM isobutyl methylxanthine, 1 mM dexamethasone, and 10µg/mL of insulin, followed by 48 h incubation in DMEM/10% FBS supplemented with 10µg/ml insulin. Then cells were maintained in DMEM/10% FBS supplemented with 2.5µg/mL insulin. At day 7, the differentiated adipocytes were treated with 5μM miR-10a-3p mimic MIM-hsa-miR-10a-3p (AUM Biotech) or the scrambled miR control and incubated for 24 h.

### RNA extraction and reverse-transcription quantitative polymerase chain reaction analysis

Total RNA was extracted from immune cells both from HFD and ND and 3T3-L1 differentiated adipocytes with the miR-10a-3p mimic and control miR (scramble) using the QIAGEN RNeasy mini kit (Cat. no. 74104). 1μg of extracted RNA from each sample was used for reverse-transcription into cDNA with an iScript cDNA synthesis kit (Cat. no. 1708891; Bio-Rad) or for miRs, a miRCURY LNA rt Kit (339340, QIAGEN, USA) according to the manufacturer’s protocols. Reverse-transcription quantitative polymerase chain reaction analysis (RT-qPCR) was performed using the iTaq Universal SYBR Green Supermix (Cat. 1725121; Bio-Rad, USA) or for miRs, the miRCURY LNA SYBR Green PCR Kit (339346, QIAGEN). Primers were purchased from Integrated DNA Technologies (IDT; Coralville, IA) and QIAGEN. The primer sequences for genes and catalog no of miR primers (from QIAGEN) are shown in **Tables 1** and **2**, respectively.

**Table 1:** The sequence of primers used to amplify the indicated gene of interest.

Gene	Forward sequence	Reverse Sequence
IL-17F	AAC CAG GGC ATT TCT GTC CCA C	GGC ATT GAT GCA GCC TGA GTG T
TGF-ß1	TGA TAC GCC TGA GTG GCT GTC T	CAC AAG AGC AGT GAG CGC TGA A
IL-6	CAC AAG AGC AGT GAG CGC TGA A	CTG CAA GTG CAT CAT CGT TGT TC
IL-1α	ACG GCT GAG TTT CAG TGA GAC C	CAC TCT GGT AGG TGT AAG GTG C
STAT-3	AGG AGT CTA ACA ACG GCA GCC T	GTG GTA CAC CTC AGT CTC GAA G
IL-1β	TGG ACC TTC CAG GAT GAG GAC A	GTT CAT CTC GGA GCC TGT AGT G
FOXP3	CCT GGT TGT GAG AAG GTC TTC G	TGC TCC AGA GAC TGC ACC ACT T
IFN-γ	CAG CAA CAG CAA GGC GAA AAA GG	TTT CCG CTT CCT GAG GCT GGA T
TNF-α	GGT GCC TAT GTC TCA GCC TCT T	GCC ATA GAA CTG ATG AGA GGG AG
IL-10	CGG GAA GAC AAT AAC TGC ACC C	CGG TTA GCA GTA TGT TGT CCA GC
Leptin	GCA GTG CCT ATC CAG AAA GTC C	GGA ATG AAG TCC AAG CCA GTG AC
CD11c	TGCCAGGATGACCTTAGTGTCG	CAGAGTGACTGTGGTTCCGTAG
iNOS	GAGACAGGGAAGTCTGAAGCAC	CCAGCAGTAGTTGCTCCTCTTC
FASN	CACAGTGCTCAAAGGACATGCC	CACCAGGTGTAGTGCCTTCCTC

**Table 2:** Primers for cDNA synthesis of the miR mimics were used in this study.

miRCURY LNA miR mimic	Qiagen Cat. no.
**hsa-miR-125b-5p**	YP00205713
**hsa-miR-21a-5p**	YP00205400
**hsa-miR-181a-2-3p**	YP00205173
**hsa-miR-125a-3p**	YP00204446
**hsa-miR-34c-5p**	YP00205659
**hsa-miR-10a-3p**	YP00205688
**hsa-miR-144-3p**	YP00204754
**hsa-miR-132-3p**	YP00206035

### Immunoblot analysis

Cells were washed twice with cold phosphate-buffered saline (PBS) and lysed with RIPA buffer supplemented with a (Thermo Fisher Scientific, Ref#78442). Cells were ruptured by pipetting, sonicated for 1 min, and incubated on ice for 30 min to ensure complete lysis. Cell debris was removed by centrifugation at 15,000 x g for 20 min. The protein concentration of the resulting supernatant was measured with a Pierce™ BCA Protein Assay Kit (ThermoFisher Scientific, Waltham, MA). Equal amounts (10μg) of protein for each sample were separated by 10% SDS (Sodium Dodecyl Sulfate)-PAGE (polyacrylamide gel electrophoresis) and transferred to PVDF membranes (620174, Bio-Rad) using a Transblot Turbo instrument (Bio-Rad). The membrane was blocked with intercept blocking buffer (92760001, LI-COR Biosciences, USA) at RT for 1 h and incubated at 4°C overnight on a shaker with primary antibodies specific for PPAR-γ (1:3,000; cat. no. 66936-1-Ig; Proteintech, Rosemont, IL), Smad3 (1:3,000; cat. no. 66516-1-Ig; Proteintech), CHOP (1:3,000; cat. no. 66741-1-Ig; Proteintech), TGF-β1 (1:200; cat. no. sc-130348; Santa Cruz Biotechnology (SCBT), Dallas, TX), C/EBPα (1:200; cat. no. sc-365318, SCBT), and β-actin (1:5,000; cat. no. 926-42212; LI-COR, Lincoln, NE). Unbound primary antibodies were removed by washing with TBST and the membranes were incubated at RT for 1 h with IRDye® 800CW-labeled goat anti-mouse (1:5000; cat. no. 926-32210; LI-COR) and goat anti-rabbit (1:5,000; cat. no. 926-32211; LI-COR) secondary antibodies. Images were taken using an LI-COR Odyssey® DLX imaging system and densitometric analyses were performed using LI-COR Image Studio Software.

### Oil Red O staining, imaging, and lipid content quantification

Differentiated 3T3-L1 adipocytes treated with scrambled miR control or miR mimic were washed twice with PBS, fixed with 4% paraformaldehyde at RT for 20 min, washed with PBS, and stained with freshly diluted Oil Red O (ORO) solution at RT for 30 min. After staining, cells were washed three times with distilled water and mounted on glass slides. Lipid droplets were observed using an Olympus BX43 bright field microscope. In another experimental replicate, ORO was extracted from the cells by washing with 2-propanol and the optical density (OD) of the extracted ORO was quantitated with a spectrophotometer (Cytation5, BioTek, USA) at a wavelength of 490 nm.

### Statistical analyses

The data were expressed as mean values ± SEM of at least three replicates per sample. Statistical analyses were performed using Student’s t-tests where *p*< 0.05 (*) and p< 0.01(**) were considered to be statistically significant. Graphical representations were generated using GraphPad Prism (GraphPad Software, Boston, MA) and Origin8 (OriginLab, Northampton, MA) software.

### Data availability

The raw and processed data reported in this paper are available in the Gene Expression Omnibus (GEO) repository under accession number GSE216944.

## Results

### Systemic and AT metabolic alteration in HFD-induced obesity

**Figure 1:**
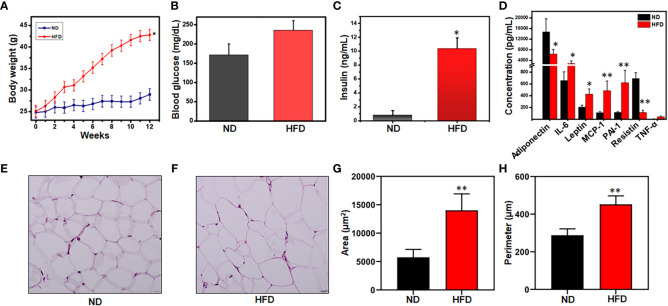
HFD alters mouse body weight, metabolic parameters, and adipocyte morphology. Mice were fed a normal diet (ND) or high-fat diet (HFD) for 12 weeks before sacrifice. **(A)** Alterations in weekly body weight were recorded. **(B)** Changes in blood glucose level were measured at the experimental endpoint. **(C)** Changes in blood insulin level were measured at the experimental endpoint. **(D)** Levels of AT lysate adipokines and cytokines were measured by Bio-Plex cytokine assay. **(E)** Representative images of H & E-stained adipose tissue (AT) from mice fed ND. **(F)** Representative images of H & E-stained AT from mice fed HFD. Sections were examined by light microscopy at a magnification of 100X. **(G)** Comparison between ND and HFD adipocyte cellular areas. The adipocyte size and perimeter area surrounded by infiltrated immune cells were larger in HFD-fed mice as compared to ND. **(H)** Comparison between ND and HFD adipocyte cell perimeters. 30 histological sections from each group were measured. Data are representative of the mean of three independent experiments. Data shown are mean values ± SEM; total n = 6. Statistically significant differences in body weight, body fat, fasting blood glucose, and plasma insulin between mice fed ND or HFD (*p<0.05) and those between adipocyte area and perimeter between HFD and ND (**p<0.01) are indicated, based on unpaired Student’s t-test.

The infiltration of immune cells into the AT is closely correlated with low-grade chronic inflammation and plays a key role in altering metabolic diseases and obesity. In AT, these infiltrated immune cells crosstalk with adipocytes to boost the obesity condition and chronic inflammation. We determined the effect of diets on body weight and other metabolic parameters in mice fed ND and those fed an HFD for 12 weeks. During this period, the body weight of mice in the HFD group increased significantly from an approximately 25g to 45g relative to that of the ND group (**Figure 1A**). Other metabolic parameters like blood glucose and insulin levels also were elevated in mice fed HFD, relative to mice fed ND (**Figures 1B, C**). We also noted that the levels of IL-6, leptin, MCP-1, PAI-1, and TNF-α in the AT were enhanced while levels of adiponectin and resistin decreased in mice in the HFD group relative to those in the ND group (**Figure 1D**). Interestingly, the adipocyte size in the HFD group was expanded two to three-fold compared to those in the ND group, which is reflected in morphometric analysis of their area and perimeter (**Figures 1E–H**). Thus, these results suggest that HFD affects the metabolic status of the system and hampers AT homeostasis. Furthermore, increased levels of IL-6, MCP-1, and leptin promote the infiltration of more inflammatory cells to the AT, thereby maintaining low-grade chronic inflammatory conditions.

### HFD-induced expression of inflammatory cytokines and transcription factors by AT immune cells

**Figure 2:**
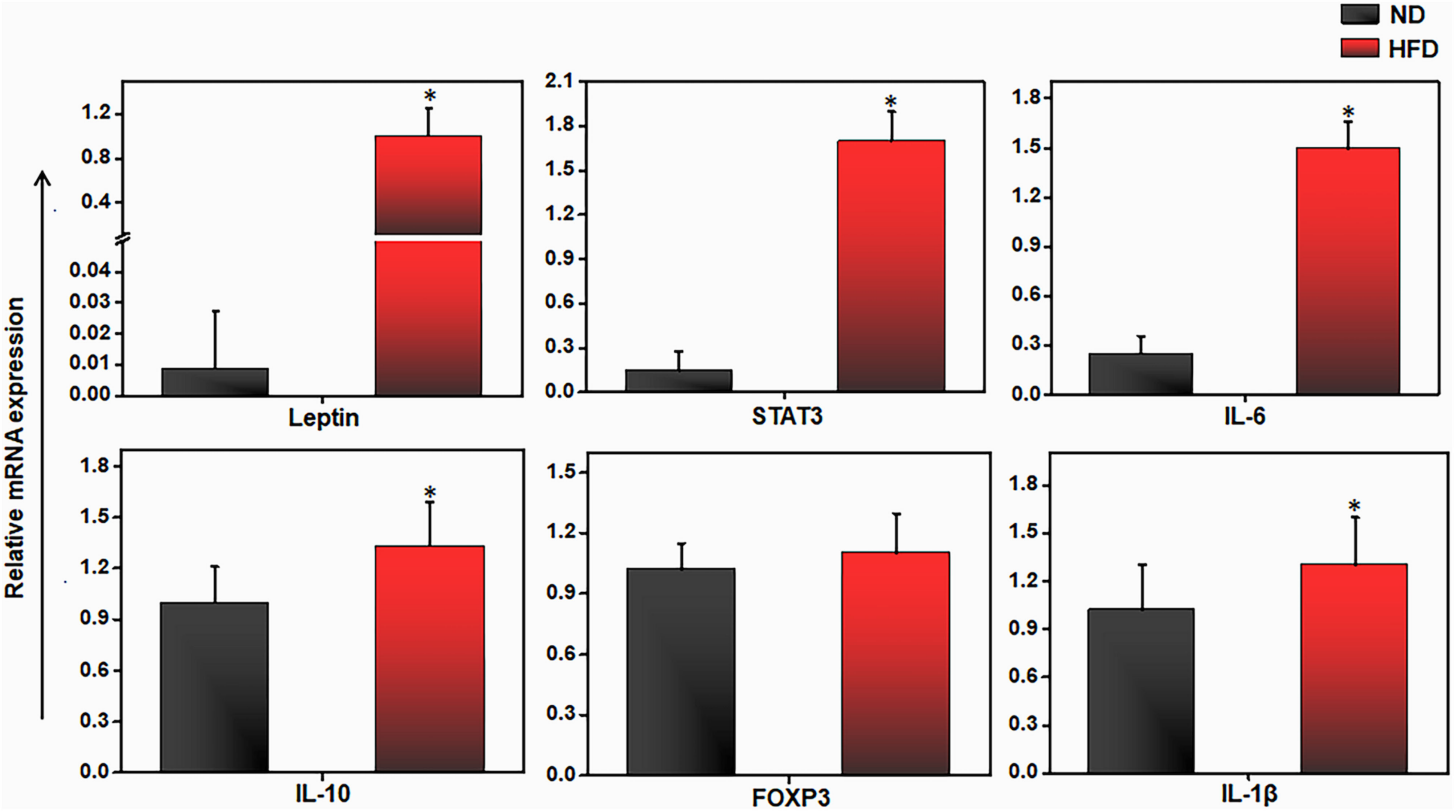
HFD alters pro and anti-inflammatory genes expression in adipose tissue. Mice were fed on HFD or ND for 12 weeks before sacrifice and AT was collected. We isolated total RNA from the AT from each group of mice, pooled, quantitated, and reverse-transcribed it into cDNA, and analyzed cDNA levels by qPCR with primers specific for the murine leptin, IL-6, IL-10, FoxP3, IL-1β and Stat3 genes. HFD mice expressed higher levels of IL-6, IL-10, leptin, and STAT3 transcripts than did mice fed ND. Vertical bars shown represent mean ± SEM. Statistically significant differences between the HFD and ND groups are indicated (*p<0.05), based on Student's T test.

Since AT-infiltrated immune cells mediate inflammation through the secretion of cytokines and chemokines (36), we analyze the expression of various cytokines and transcription factors in AT immune cells from mice fed ND or HFD. We observed that the expression of leptin, STAT-3, IL-6, and IL-10 was significantly upregulated in the HFD group, relative to the ND group (**Figure 2**). We also observed a slight increase in the expression of forkhead family transcription factor p3 (FoxP3) and IL-1β in the AT from mice fed HFD as compared to those fed ND. These results suggest dysregulation of proinflammatory and anti-inflammatory cytokine expression in mice fed HFD as compared to those fed ND. To explore the underlying factors responsible for the cytokine imbalance during HFD feeding, we evaluated whether the miR profile in AT immune cells plays any role during HFD-induced obesity.

### Differential expression of miRs in AT immune cells during obesity

**Figure 3:**
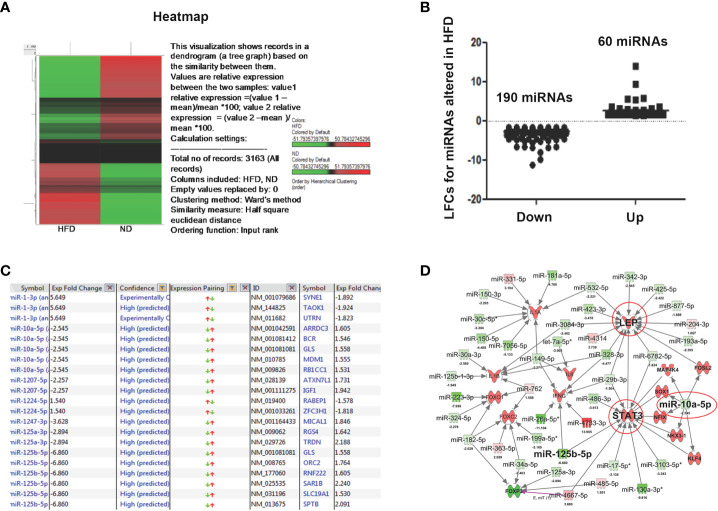
Microarray analysis of differentially expressed miRs from AT-resident immune cells from mice fed ND or HFD. Changes in unsupervised hierarchical clustering of differentially expressed miRs in HFD-induced obesity versus ND-fed group controls between the AT-derived immune cells reveal the presence of divergent pathways. **(A)** Heatmap shows unsupervised hierarchical clustering of differentially expressed miRs. Upregulated miRs are shown in red, while downregulated miRs are shown in green. **(B)** A plot of normalized Log2-fold change of 60 upregulated and 190 downregulated miRs. The linear fold change 1.5 cut-off value was considered. **(C)** List of highly predicted altered miRs with fold change values indicated. **(D)** IPA shows immunomodulatory target pathways and miRs that interact with known direct and indirect targets of interest.

In obesity, the expression levels of several miRs appear to be altered in the AT (37). To further explore the dysregulation of miRs in the AT immune cells during obesity, we performed miR array analysis in AT immune cells of mice fed ND or HFD for 12 weeks. The resulting heat map of hierarchical clustering analysis depicted a subset of differentially expressed miRs (**Figure 3A**). After global normalization of the raw data, we identified differentially expressed miRs in which 60 miRs that were significantly (>1.5-fold) upregulated and 190 that were significantly (>1.5-fold) downregulated in HFD fed mice, compared to mice fed ND (**Figure 3B**). Highly predicted altered miRs in obesity, including miR-10a and miR-125, are shown in **Figure 3C** with their respective fold changes. Further, deep analysis using IPA software was used to delineate a possible miRs-mediated pathway to alter obesity. The altered miRs were largely associated with genes related to metabolic disease. Of the miRNAs that were upregulated or downregulated by > 1.5-fold in response to HFD, 2 had a direct association with an inflammatory pathway, adiposity, and immune modulation target. To infer the function of these miR targets, we performed an IPA for the miRs that showed ectopic expression related to metabolism and inflammation (**Figure 3D**). The analysis demonstrated that miR-10a directly targeted leptin and STAT-3, and indirectly targeted IL-1β and Foxp3, all of these regulate adipogenesis (38). Taken together, these data suggest that miRs expressed in AT immune cells during obesity directly target the proinflammatory genes *via* different pathways. Our next step was to confirm that the expression of these miRs was altered in AT immune cells during HFD-induced obesity.

### Alteration of miR expression in AT immune cells

**Figure 4:**
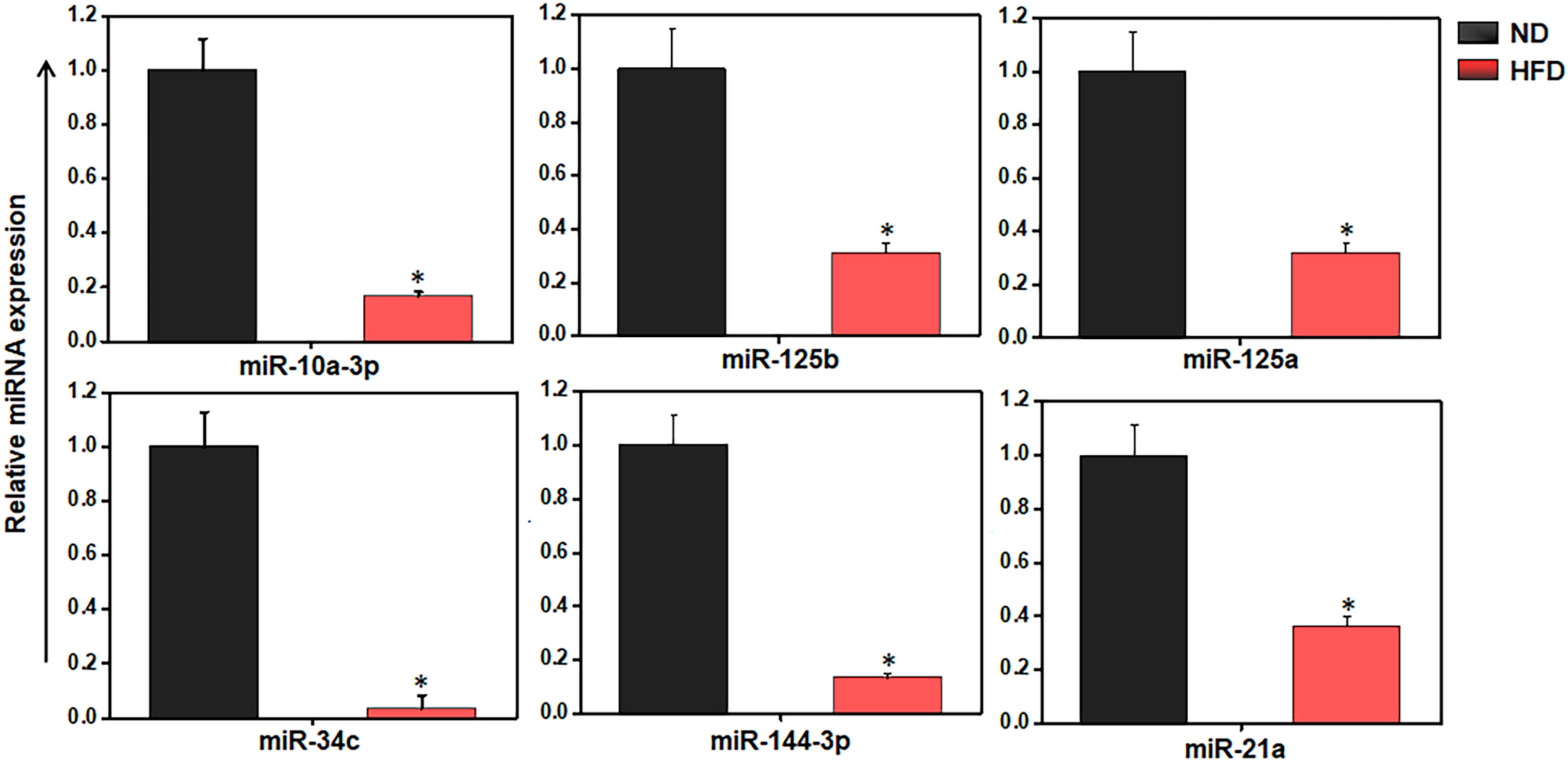
HFD alters the inflammation and metabolism-related miRs expression in AT-resident immune cells. Validation of selected eight miRs in AT immune cells from ND and HFD-fed mice by RT-qPCR analysis. The results verify the downregulation of miR-10a, -125b, -125a, -34c, -144, -21a, and -132 are shown are mean values ± SEM. Statistically significant differences between the HFD group and the ND group are indicated (*p<0.05), using the Student’s t-test.

We used RT-qPCR to further analyze the expression of different miRs in the AT immune cells of mice fed HFD versus ND. We observed the downregulation of miR-10a-3p, miR-34c-5p, miR144-3p, miR-21a-5p, miR-125a-3p, and miR-125b-5p (**Figure 4**). The similar trends were detected by both RT-qPCR and microarray analysis confirmed the importance of miRs in AT inflammation during HFD-induced obesity. We selected miR-10a for further analysis in light of this promising data, its high degree of prediction by software analysis, and previous literature on the differential roles of miR-10a in the regulation of TGF-β (27 , 28) and its anti-inflammatory properties (39–41). We then used the miR mimic approach as a mean to restore the function of the downregulated miR-10a in AT-derived immune cells from HFD-fed mice and to overexpress of miR-10a-3p in differentiated 3T3-L1 adipocytes.

### miR-10a-3p mimic attenuates the expression of inflammatory genes and signaling pathways in AT immune cells derived from HFD-fed mice

**Figure 5:**
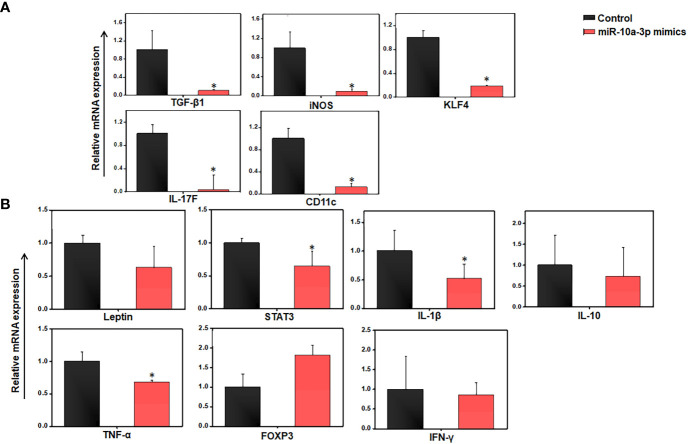
Differences in gene expression between control and miR-10a-3p mimicked AT resident immune cells from HFD mice in *ex vivo* culture. Mice were fed a high-fat diet (HFD) for 12 weeks before sacrifice and AT immune cells were isolated. These cells were seeded in triplicate in 12 well plates at a density of 0.5x10^6^/well, treated with 5 μM miR-10a mimic MIM-hsa-miR-10a-3p (AUM Biotech, Philadelphia, PA, USA) or a scrambled miR control, and incubated for 24 h. **(A)** Total RNA from each group was isolated, pooled, quantitated, reverse-transcribed into cDNA, and analyzed by qPCR with primers specific for TGF-β1, PPAR-γ, KLF4, IL-17F, CD11c, and iNOS. **(B)** Changes in adipokine levels were quantitated by RT-qPCR using primers specific for Leptin, STAT3, IL-1β, IL-10, TNF-α, IL-6, IFN-γ, and FOXP3. Shown are mean values ± SEM. Statistically significant differences between the HFD group and the ND group are indicated (*p<0.05) using the Student’s t-test.

In light of the evidence that miR-10a expression is downregulated in AT immune cells from HFD-fed mice (**Figures 3C**, **4**), we isolated AT immune cells from HFD-induced obese mice after 12 weeks and treated them for 24 h with scrambled control miRs or a synthetic miR-10a-3p mimic. When we used RT-PCR to analyze the expression of various proinflammatory and anti-inflammatory genes, we found that relative to controls, AT immune cells treated with the miR-10a-3p mimic exhibited significantly reduced expression of TGF-β1, KLF4, IL17F, CD11c, and inducible nitric oxide synthase (iNOS) (**Figure 5A**). We also observed that the expression of IL-6 and FoxP3 was significantly increased while that of leptin, STAT-3, IL-1β, IL-10, TNF-α, and IFN-γ was decreased in cells treated with the miR-10a-3p mimic, compared to their levels in cells treated with the control scrambled miR (**Figure 5B**). These data suggest that treatment with the miR-10a-3p mimic checked the expression of various well-known inflammatory genes and signaling pathways that suppress AT inflammation. However, whether this reduction in inflammatory pathways reduced adiposity and by what mechanism remains unclear. Thus, this fascinating result encouraged us to extend our work to determine the effect of the miR-10a-3p mimic on adipocyte biology and function.

### miR-10a-3p mimic impedes inflammation and lipid accumulation in cultured 3T3-L1 adipocytes

**Figure 6:**
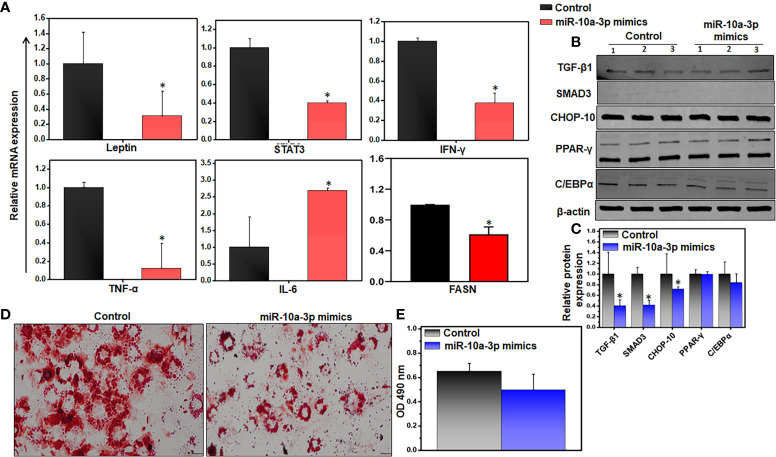
Variations of inflammatory and adipogenic markers of miR-10a-3p mimicked 3T3-L1 adipocytes. We used the 3T3-L1 pre-adipocyte cell line (ATCC-CL-173) to confirm the effect of the miR mimics. Differentiation of 3T3-L1 pre-adipocytes into adipocytes was induced using our laboratory’s standard protocol. The resulting adipocytes were treated with 5 μM miR-10a mimic MIM-hsa-miR-10a-3p (AUM Biotech) or a scrambled miR control and incubated for 24 h. **(A)** Changes in expression of inflammatory genes (Leptin, STAT-3, IFN-γ, TNF-α, and FASN). **(B, C)** Representative immunoblot and its quantitation for relative levels of TGF-β, Smad3, CHOP-10, PPAR-γ, and C/EBPα. **(D)** Representative images of 3T3-L1 cells stained with ORO. The decrease in lipid accumulation in adipocytes was noticed in the miR-10a-3p mimic treated group as compared to scramble miR control. **(E)** Quantitation of lipid staining in ORO-stained 3T3-L1 cells by optical density. Data are representative of the mean of three independent experiments; shown are mean values ± SEM; total n = 6. Statistically significant differences between cells treated with the miR-10a mimic and the scrambled miR control are indicated (*p< 0.05), based on an unpaired Student’s t-test.

We previously showed that miRs are involved in crosstalk between immune cells and adipocytes (42). miRs can be exported from one cell to another and function in a paracrine fashion. These data led us to wonder whether miR-10a expressed in AT resident immune cells might affect neighboring adipocytes. To limit the effects on adipocytes, we used cultured differentiated 3T3-L1 adipocytes rather than AT to test the effect of treatment with the miR-10a-3p mimics *vs.* the scrambled miR control on gene expression by RT-qPCR analysis. The results suggested that treatment of 3T3-L1 adipocytes with the miR-10a-3p mimic led to significant downregulation of the pro-inflammatory and metabolic genes, namely leptin, STAT3, IFN-γ, TNF-α, and FASN, and upregulation of IL-6, relative to the control (**Figure 6A**).

Since expression of TGF-β1 is positively correlated with obesity and TGF-β1 transmits its signals *via* the transcriptional factor Smad3 (23), we examined the effect of treatment with the miR mimic on the TGF-β/Smad3 signaling pathway during obesity. We treated differentiated 3T3-L1 adipocytes with the miR-10a-3p mimic for 24 h and analyzed the expression of downstream proteins responsible for adipogenesis by western blot analysis. We observed decreased levels of the TGF-β1, Smad3, and CHOP-10 proteins, but no change in PPAR-γ levels, and only a slight reduction in C/EBPα protein levels in cells treated with the miR-10a-3p mimic, relative to the control (**Figures 6B, C**). We also analyzed the effect of the miR-10a-3p mimic on an accumulation of lipids by staining the treated 3T3-L1 adipocytes with ORO and examining the stained cells by light microscopy. We observed a decreased lipid accumulation in the miR-10a-3p mimic-treated group relative to the control group (**Figure 6D**). To confirm this result, we eluted the ORO dye from the treated, stained adipocytes and measured the OD by spectroscopy, where the OD directly indicates lipid deposition in the adipocytes. Remarkably, the adipocytes treated with the miR-10a-3p mimic contained less lipid than the scrambled miR controls (**Figure 6E**), a finding that was consistent with our observation that FASN expression levels were reduced under these conditions (**Figure 6A**). These observations strongly suggest that treatment of cultured differentiated 3T3-L1 adipocytes with a miR-10a-3p mimic reduced lipid accumulation and thus that miR-10a-3p not only alters inflammatory gene expression in AT immune cells but also mediates expression of adipogenic genes in adipocytes.

## Discussion

Obesity and associated metabolic disorders are becoming major global healthcare challenges. Obesity-induced inflammation also affects metabolic diseases and paves the way for many inflammatory diseases by maintaining low-level chronic inflammation (43). Current interventions are insufficient to prevent premature morbidity and mortality arising from obesity, which can modify and worsen many other diseases including cancer. Thus, there is an urgent need to understand the pathogenesis and find a way to limit the overexpression of inflammatory genes and pathways as a means of developing a successful therapeutic approach for the treatment of inflammation-related metabolic disorders and obesity. Current studies suggest that miRs regulate these changes in obese AT and can accelerate or inhibit adipocyte differentiation and chronic inflammation, suggesting that miRs may provide novel therapeutic targets for obesity (25). Therefore, in this study, we investigated the mechanisms by which miR-10a-3p mediates obesity, adiposity, and AT inflammation. We found that in both *ex vivo* and *in vitro*, treatment with a miR-10a-3p mimic reduced the expression of the murine leptin, STAT3, TGF-β1, Smad3, and CHOP-10 genes. Furthermore, our observations that treatment with the miR-10a-3p mimic led to a significant decrease in the expression of IL-17F and FASN, decreased lipid accumulation, and an increase in the expression of FoxP3 suggest a mechanism for the miR-10a-3p mediated suppression of adiposity. These data support our interpretation that during obesity the miR-10a-3p mimic significantly reduced expression of systemic and AT inflammatory cytokines in AT immune cells and reduced excess lipid accumulation in adipocytes, in part through the TGF-β1/Smad3 mediated pathway. Hence, miR-10a-3p mimic serves as a potential therapeutic tool to modulate the function of adipose tissue and thereby control obesity.

Obesity is the manifestation of an excessive storage of energy that could potentially be ameliorated by activating the body’s dormant energy-burning system. An increase in body weight, blood glucose, and insulin levels are the hallmarks of obesity progression (44). Our results indicate the occurrence of obesity in the HFD-fed mouse group relative to the mice fed ND. Leptin is an important mediator of metabolism and inflammation (45). In the present study, we observed the overproduction of leptin, IL-6, MCP-1, PAI-1, and TNF-α, indicating the presence of increased inflammation in the HFD group relative to the ND group. We also observed the decreased expression of adiponectin and resistin in the HFD-fed mice, supporting the onset of obesity and corroborating the results of a previous study (46). Furthermore, a 2 to 3-fold increase in adipocyte size in HFD-fed mice illustrates their hypertrophy relative to the adipocytes from ND-fed mice. Interestingly, the expression of TNF-α, IL-6, and MCP-1 is positively and that of adiponectin is negatively correlated with adipocyte size (47, 48). Adipocyte hypertrophy is also highly correlated with inflammation. Taken together, our data suggest that HFD induces metabolic dysregulation by increasing the expression of proinflammatory cytokines and adipocyte size by leptin and TNF-α.

We also confirmed the existence of AT inflammation with our observation of increased expression of IL-6, IL-1β, leptin, and STAT3 in the AT of HFD-fed mice relative to ND-fed mice. Leptin induces the production of IL-6 and IL-1β (49, 50) and signaling *via* the JAK2-STAT3 pathway (51). Paradoxically, we also observed increased expression of the anti-inflammatory cytokine IL-10 in HFD-fed mice relative to ND-fed mice. Although IL-10 is mostly negatively correlated with obesity, the AT of obese individuals exhibits increased IL-10 production, which may serve to regulate the inflammatory state (52). IL-10 secreted from regulatory T (Treg) cells suppresses white adipose tissue (WAT) browning (53) and induces obesity. Thus, data at our disposal suggests in part that the elevated level of IL-10 is supported by higher expression of FoxP3 in Treg cells after treatment with the miR-10a-3p mimic. However, a detailed investigation of HFD-induced obesity and metabolic dysregulation will be required to address this apparent discrepancy in the expression of pro- and anti-inflammatory cytokines and transcription factors.

Mounting evidence suggests the dysregulation of miRs in obesity (37, 54, 55). In this study, we detected a total of 250 miRs that were dysregulated in the AT immune cell population in HFD-fed mice relative to ND-fed mice. Based on pathway analysis of metabolic and immune dysregulation (34, 56–59), we selected eight miRs (miR-10a-3p, miR-125a, miR125-b, miR-34c, miR144-3p, and miR-21a) for further investigation. For the current study, we focused on miR-10a based on the results of IPA analysis that miR-10a expression was relative to that of two metabolic and inflammatory genes, namely leptin and STAT3. We observed an increase in leptin and STAT3 expression in AT of HFD-fed mice relative to ND-fed mice. Furthermore, our RT-qPCR results confirmed the downregulation of miR-10a in the AT immune cells from HFD-fed mice, highlighting the protective role of miR-10a in obesity and related chronic inflammation in the AT. Interestingly, expression of the miR-10a-3p mimic reverses the alteration of leptin and STAT3 expression and reduces AT inflammation, which encourages us to dig deeper for mechanistic outcomes in our future studies.

Interestingly, anti-inflammatory roles for miR-10a are reported in rheumatoid arthritis and inflammatory bowel disease (IBD) (41, 60). Our *ex vivo* findings suggest that the expression of most of the inflammatory markers (iNOS, leptin, TNF-α, IL-17F, TGF-β, and CD11c) in AT resident immune cells was attenuated after treatment with the miR-10a-3p mimic compared to those treated with the scrambled miR control. A subset of dendritic cells (DCs) that express macrophage markers in obese mice induces differentiation of Th17 cells (61). This study established that the gain of miR-10a function has the potential to regulate the activity of both IL-17F and DC cells in obesity. In the past, similar outcomes were obtained in the treatment of IBD with miR-10a, which inhibits Th17 and DC cells (60). KLF4, which is responsible for M2 macrophage polarization (Liao et al., 2011), is a direct target of miR-10b (62, 63). In the current study, we also observed a decrease in KLF4 expression after treatment with a miR-10a-3p mimic. A time point *in vivo* study will be required to examine the function of miR-10a in relation to KLF4 in the context of inflammatory macrophages.

miR-10a negatively regulates IL-10 production by CD4^+^ T cells (64). While the current study examined the effect of the miR-10a-3p mimic on AT immune cells, further work will be required to determine whether treating T cells with miR-10a-3p mimics will inhibit their production of IL-10 production. Treg cells are an important regulator of the inflammatory response and their population is reportedly decreased during obesity (65). The role of Treg cells in inflammation is regulated by miR-10a, which stabilizes FoxP3 (66, 67). Our observation that treatment of AT immune cells from HFD-induced obese mice with a miR-10a-3p mimic induced the expression of FoxP3 further supports these previous findings. Taken together, treatment with miR-10a-3p mimics reduced inflammation in AT resident immune cells *via* mechanisms that downregulate the inflammatory response while simultaneously activating Treg cells.

The direct targets of miR-10a include Fos-related antigen 2, KLF4, SRY-box transcription factor 1, and nuclear factor I as shown in **Figure 3D**. It has been shown that FOSL2 positively regulates TGF-β1 signaling in non-small cell lung cancer (68). Further FOSL2 also promotes leptin gene expression in human and mouse adipocytes (69). It has been well established that KLF4 regulates macrophage polarization (70) and plays a role in obesity. Our study suggests that miR-10a overexpression targets FOSL2 and mediates leptin and TGF-β1 expression to suppress the adipogenesis and inflammatory response in AT. Further, our study miR-10a also target KLF4 to mediate STAT3 and other inflammatory markers in adipocyte. However, a further detailed study is required for a precise direct target of miR-10a to suppress adipogenesis and inflammatory response in AT.

Inflammation and metabolism are tightly interlinked and regulate each other (71). Our data indicate that miR-10a-3p plays a role in inflammation in the context of obesity, which led us to determine the effects of the miR-10a-3p mimic on adiposity and adipocyte function. Towards this, both miR-10a-5p and miR-10b-5p regulate AT remodeling and differentiation by targeting the expression of RAR-related orphan receptor alpha and apolipoprotein L6, respectively (57, 72). Our *in vitro* findings showed that miR-10a-3p mimic reduced the expression of metabolic and inflammatory genes, including leptin, STAT3, TNF-α, and IFN-γ in differentiated 3T3-L1 adipocytes. Further, our previous study demonstrated overexpression of TGF-β in HFD-induced obesity (54). To this end, we observed that the miR-10a-3p mimic downregulated TGF-β1 *ex vivo* in AT immune cells from HFD-fed mice and *in vitro* in cultured 3T3-L1 adipocytes. There is mounting evidence that miR-10a targets TGF-β1 in renal and hepatic fibrosis, (27, 28). The downstream partner of the canonical TGF-β1 pathway is Smad3 (73), which blocks the activity of C/EBPβ and C/EBPδ in transcription (74). C/EBPβ and C/EBPδ induce transcription of PPAR-γ (75) and PPAR-γ and C/EBPα function cooperatively to stimulate transcription in the later stage of adipocyte differentiation (76). CHOP-10 is a repressor of adipogenic differentiation that blocks C/EBPβ binding to DNA (77). Interestingly, our results demonstrated that the miR-10a-3p mimic reduced the expression of the TGF-β1, Smad3, and CHOP-10 proteins but did not affect the expression of PPAR-γ and C/EBPα. Thus, the miR-10a-3p mimic promoted adipocyte differentiation by inhibition of TGF-β1/Smad3 and CHOP-10. Differentiated 3T3-L1 adipocytes treated with the miR-10a-3p mimic exhibited reduced lipid accumulation, perhaps due to the downregulation of FASN, which catalyzes the formation of long-chain fatty acids. The recent report demonstrated that miR-10a-5p targets FASN (78), which supports the results of our study. At this point, we believe that adipocyte hypertrophy is more threatening than hyperplasia as it causes lipotoxicity and stimulates the immune response (79).

In summary, the present study suggests that miR-10a-3p mimics reduce AT inflammation and maintain AT homeostasis by inducing healthy adipocyte differentiation instead of hypertrophy. Thus, regaining the function of miR-10a-3p might represent a potential therapeutic approach for treating obesity and associated AT inflammation and metabolic diseases. Overall, miR-10a-3p functions as both an anti-inflammatory and an anti-obesity agent, which is promising for the future development of novel treatments for obesity and metabolic diseases.

## Data availability statement

The datasets presented in this study can be found in online repositories. The names of the repository/repositories and accession number(s) can be found below: https://www.ncbi.nlm.nih.gov/geo/, GSE216944.

## Ethics statement

All animal experimentation was performed under protocols (20-0162) approved by the University of Tennessee Health Science Center (UTHSC) Institutional Animal Care and Use Committee (IACUC).

## Author contributions

SK, MM, and AR: performed the majority of the experiments, and data analysis, wrote the first draft of the manuscript, and made all the figures. AB: provided the cell lines, helped in data analysis, and edited the manuscript. US: conceived the idea, design the experiments and edited the manuscript. All authors contributed to the article and approved the submitted version.
